# Retraction Note: LncRNA SOX21-AS1 accelerates endometrial carcinoma progression through the miR-7-5p/RAF1 pathway

**DOI:** 10.1186/s12957-023-03201-8

**Published:** 2023-09-30

**Authors:** Meng Sun, Dongxu Chen, Youguo Chen, Yibo Wu

**Affiliations:** 1 Department of Gynecology, 1St Affiliated Hospital, Soochow University, Gusu District, 188#, Shizi Street, Suzhou, 215000 Jiangsu China; 2 Department of Gynecology, Affiliated Hospital of Jiangnan University, Wuxi, 214000 Jiangsu China; 3 Department of Nuclear Medicine, Wuxi Branch of Ruijin Hospital, Wuxi, 214000 Jiangsu China; 4 Human Reproductive and Genetic Center, Affiliated Hospital of Jiangnan University, 200 Huihe Road, Wuxi, 214000 Jiangsu China

**Retraction Note: World J Surg Oncol 21, 217 (2023)**

**https://doi.org/10.1186/s12957-023-03114-6**

The authors have retracted this article because they found that the presented data had been fabricated.

All authors agree to this retraction.

